# Widefield optical coherence tomography by electro-optical modulation

**DOI:** 10.1364/BOE.540278

**Published:** 2024-10-28

**Authors:** Dorian R. Urban, Pavel Novak, Miguel A. Preciado, Tom Vettenburg

**Affiliations:** 1 Optos PLC, Queensferry House, Enterprise Way, Dunfermline KY11 8GR, Scotland, UK; 2 School of Science and Engineering, University of Dundee, DD1 4HN, Scotland, UK; 3 Centre for Medical Engineering and Technology, Dundee DD1 4HN, Scotland, UK

## Abstract

Optical coherence tomography (OCT) is a unique imaging modality capable of axial sectioning with a resolution of only a few microns. Its ability to image with high resolution deep within tissue makes it ideal for material inspection, dentistry, and, in particular, ophthalmology. Widefield retinal imaging has garnered increasing clinical interest for the detection of numerous retinal diseases. However, real-time applications in clinical practice demand the contrast of swept-source OCT at scan speeds that limit their depth range. The curvature of typical samples, such as teeth, corneas, or retinas, thus restricts the field-of-view of fast OCT systems. Novel high-speed swept sources are expected to further improve the scan rate; however, not without exacerbating the already severe trade-off in depth range. Here, we show how, without the need for mechanical repositioning, harmonic images can be rapidly synthesized at any depth. This is achieved by opto-electronic modulation of a single-frequency swept source laser in tandem with tailored numerical dispersion compensation. We demonstrate experimentally how real-time imaging of highly-curved samples is enabled by extending the effective depth-range 8-fold. Even at the scan speed of a 400 kHz swept source, harmonic OCT enables widefield retinal imaging.

## 1. Introduction

Optical Coherence Tomography (OCT) produces high-resolution cross-sectional images. Axial sections can be acquired by illuminating a spot in the sample with broadband, temporally incoherent, light. The image of the axial section is then reconstructed by analyzing the interference of the back-scattered light with a delayed reference beam. Sequentially-recorded axial sections, or A-scans, are often combined into two or three-dimensional images, known as B or C-scans. Since its development in the early 1990s, OCT has been recognized as a state-of-the-art, non-invasive, biological imaging technique thanks to its ability to produce volumetric images at a resolution on the order of microns. Today, the primary application of OCT is in ophthalmology, where it is instrumental for the detection and assessment of diseases such as age-related macular degeneration, glaucoma, and diabetic retinopathy [1–3].

The effectiveness of widefield and ultra-widefield retinal imaging, corresponding to >60^∘^ and >110^∘^ field-of-view, respectively, is increasingly recognized for early disease detection and consequent monitoring in clinical practice [4,5]. E.g., it permits simultaneous capture of macular regions and periphery features in a single image. Retinal OCT systems often rely on patient fixation to image in the periphery, making it difficult to image the same area consistently [6–8]. The advantage of widefield OCT imaging has been recognized for early detection of conditions such as diabetic retinopathy [9]. More advanced OCT approaches such as OCT Angiography have also been found to benefit from widefield capability [10]. Several ultra-widefield imaging modalities, including fundus photography, autofluorescence, and angiography, are already commercially available [11]. Whilst prototype OCT demonstrations approach the ultra-wide-field region [6,12,13]; fast commercial OCT devices remain restricted to a retinal field-of-view below $60^{\circ }$ . The curvature of typical samples such as the retina introduces an optical path difference beyond the temporal coherence length of the broadband illumination used in conventional OCT [12].

Although time-domain OCT can in principle have any axial range, mechanical scanning renders it impractically slow for *in vivo* measurements. Life imaging applications as in ophthalmology require real-time measurements to minimize discomfort as well as motion artefacts during involuntary saccades. Instead of relying on time-consuming mechanical scanning, Fourier-domain OCT reconstructs the complete A-scan as the Fourier transform of the spectrum. Moreover, Fourier-domain systems have an important advantage in terms of speed and sensitivity [14–16].

**Fig. 1.**
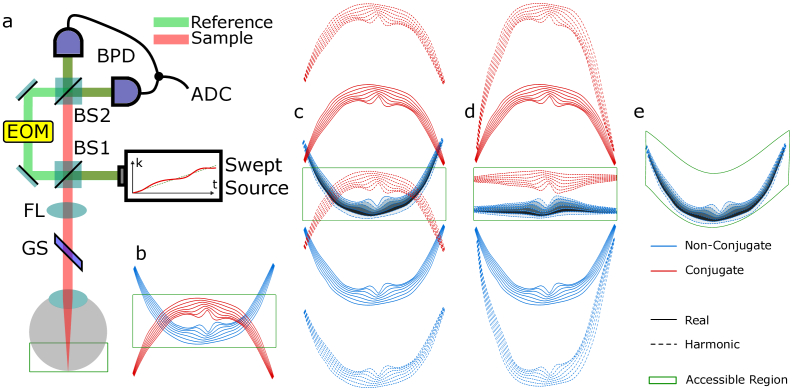
Harmonic Optical Coherence Tomography. **a.** Harmonic OCT system layout: BS1, 80:20 beamsplitter; BS2, 50:50 beamsplitter; FL, focusing lens; GS, galvo-scanner; EOM, electro-optical modulator; BPD, balanced photo-detector; ADC, analog-to-digital converter. **b.** Conventional OCT imaging, notice we cannot image the entire retina due to the curvature of the surface. **c.** After de-tuning the reference arm length to move the real image out of the accessible region, the optical phase of the reference arm is rapidly modulated, this leads to generation of sideband harmonics above and below the frequency of the real image, only the low frequency sideband is imaged, whilst other images are filtered out. **d.** The phase modulation frequency is varied as a function of lateral position, we can use this principle to cancel out the curvature of the retina, and therefore keep the entire surface in frame during acquisition. **e.** The curvature of the image is reconstructed post-acquisition, the entire retina can be recovered this way, alternatively, we can say that the imaging depth limit has been adjusted to conform to the curvature of the retina.

The axial range of Fourier-domain OCT systems is however restricted by the presence of a spurious conjugate image (cfr. Fig. 1(b)). The first implementations of Fourier-domain OCT were predominantly based on spectrometers [17], though its finite spectral resolution limits its sensitivity and effective imaging depth range. Swept-source OCT systems address this issue by recording the spectrum in time-sequence whilst rapidly scanning the optical frequency of a narrow linewidth laser.

Swept-source OCT trades-off axial range for imaging speed. This is a problem for widefield OCT systems due to the large variations in depth across the field-of-view. Yet, life imaging demands the speed and signal-to-noise contrast of swept source OCT. Several techniques have been developed with the aim of extending the axial range of OCT: exploiting the coherence revival effect exhibited by short cavity lasers [18]; utilizing aliasing as a way of data compression [19,20]; and the use of multiple reference arms [21,22]. Coherence revival can cause ghost artefacts in the image due to reflections from internal optical components of the OCT system. Methods based on aliasing require complex frequency comb laser sources, making them complex and not suitable for clinical applications; incorporating multiple reference arms is a robust, though bulky and expensive solution. Electronic frequency shifting between photo-detection and data acquisition extends the OCT range with modest analog-to-digital conversion bandwidth [23]. This approach, devoid of mechanical movement, uses a linear-in-wavenumber swept-source laser to prevent spectral broadening. However, the axial shifting range of the system is still limited by the bandwidth of the photo-detector.

The trade-off between speed and axial range can also be relaxed by dynamically adjusting the imaging depth to conform to the contour of the sample. This maximizes the utilization of the available bandwidth. One technique involves rapid movement of the reference mirror during scanning to track the curvature of the retina [24,25]. A recent approach pre-scans the human oral cavity to generate a contour map to generate a scanning pattern tailored to the sample. While this improved fidelity at longer axial distances, it is very time-consuming due to the slow movement of the mechanical reference arm [26].

Here, we introduce Harmonic Optical Coherence Tomography to enable widefield imaging at the speed and contrast of swept-source OCT. The technique builds on three key innovations. We employ radio-frequency electro-optic phase modulation of the reference arm to produce a harmonic image at a programmable depth. Secondly, novel Micro-Electromechanical Vertical Cavity Surface Emitting Lasers (MEMS-VCSEL) are used. With coherence lengths in excess of 150m , these maintain sensitivity throughout the depth range of interest to biomedical imaging [27–29]. Finally, we found that the unavoidable variations in the laser sweep rate lead to a new source of phase errors in the harmonic component. We eliminate the dispersion-like blur by numerical dispersion compensation with the Pseudo Wigner-Ville distribution.

Harmonic OCT uses heterodyne detection with electro-optic phase modulation. This is reminiscent of frequency-shifting for complex-conjugate removal [30]; however, Harmonic OCT counteracts an optical path difference with the corresponding frequency offset to enable electronic depth control. In contrast to other methods of dynamic depth adjustment [24,25], our technique requires no mechanical motion. Unlike electronic domain frequency-shifting methods [23], our approach induces an optical frequency shift prior to the photo-detector, thereby eliminating any detector-bandwidth depth-restriction. Harmonic OCT is complementary to contour tracking [26], as well as axial motion compensation [25,31,32]. It allows near-instantaneous imaging depth adjustments, faster than the 400kHz A-scan rate.

In what follows we introduce the concept of Harmonic Optical Coherence Tomography and its image formation process. This hybrid opto-digital imaging technique requires the joint design of the electro-optical modulation and the numerical post-processing. In Section 3 we describe how we retrofitted a commercial OCT platform with a state-of-the-art MEMS-VCSEL source. We discuss the need for numerical dispersion compensation and show its practical implementation. Finally, in Section 4 we showcase Harmonic OCT for widefield imaging of highly-curved samples: the concave retina of a model eye and a convex biological sample. We start by showing how *μ*s-scale depth-adjustments enable an effective 8-fold extension of the axial range. Microsecond-scale depth adjustments within a fast B-scan then enable tracking of the curved retina surface and rapid high-contrast widefield imaging.

## 2. Fast electro-optical depth selection

Fourier-domain OCT systems compute an axial image as the inverse Fourier transform of the interferogram spectrum. While this is much faster than time-domain techniques, its spectral resolution does limit the imaging depth range. This is also true for swept-source OCT systems, where the spectral resolution is proportional to the photo-detector bandwidth and its sampling rate. Moreover, since the axial image is computed from the spectral intensity, a spurious conjugate image can overlap with the non-conjugate image (Fig. 1(b)). To see this, consider the image of a single scatterer at an axial distance, $z_{s}$ , in a sample with a background refractive index, n . Provided that $|z_{s}|/c$ is well within the coherence time of the source, the intensity reaching the two balanced photodetectors is proportional to $I_{\pm }(k)\propto A_{r}^{2}+A_{s}^{2}\pm 2A_{r}A_{s}\cos\left(2nkz_{s}\right)$ . Usually, the amplitude of the reference, $A_{r}$ , is much larger than the scattered amplitude, $A_{s}$ . The intensity can be split into the interference term, $2A_{r}A_{s}\cos\left(2nkz_{s}\right)$ and the background, $A_{r}^{2}+A_{s}^{2}$ . The interference term is used to reconstruct the axial image using the inverse Fourier transform in the variable 2nk . The resulting axial image can be seen to be proportional to the sum of two Dirac delta functions, $A_{s}\delta \left(z-z_{s}\right)+A_{s}\delta \left(z+z_{s}\right)$ . The former term corresponds to the true image of the scatterer, while the latter is its spurious conjugate image.

With Harmonic OCT, we overcome these limitations through a combination of heterodyne detection and digital dispersion compensation. We replace the function of the delay stage by electro-optical phase modulation, $A_{m}\sin(\omega_{m}t)$ , of the reference beam in a swept-source OCT system (Fig. 1(c)). This produces a series of harmonics in the optical field, one of which is shifted exactly by the modulation frequency, $\omega_{m}$ . In swept-source OCT, an optical frequency shift can be thought of as virtually delaying or advancing the reference, not unlike the mechanical delay line in conventional systems. The interference spectrum of the $p^{th}$ harmonic as a function of wavenumber, k=ω(t)/c , is proportional as follows

(1) $$I_{p}(k)\propto \cos\left[\phi \left(t-2\frac{n}{c}z_{s}\right)-\phi (t)-p\omega_{m}t\right]\approx \cos\left[2nz_{s}k+p\omega_{m}t-2\frac{n^{2}}{c^{2}}\alpha (t)z_{s}^{2}\right],$$

where we used a second order Taylor expansion in time of the reference beam phase, $\phi (t+\Delta t)\approx \phi (t)+\omega (t)\Delta t+\frac{1}{2}\alpha (t){\left(\Delta t\right)}^{2}$ . Its first and second derivatives are denoted by ω and α , respectively. We can express time, $t=\omega^{-1}(ck)$ , as a function of wavenumber, k ; and use the first order approximation of $ck=\omega (t_{0}+\Delta t)\approx \omega (t_{0})+\alpha (t_{0})\Delta t$ , at the sample time, $t_{0}$ , to get,

(2) $$I_{p}(k)\underset{∼}{\propto }\cos\left\{2nz_{s}k+p\omega_{m}\left[t_{0}+\frac{ck-\omega (t_{0})}{\alpha (t_{0})}\right]-2\frac{n^{2}}{c^{2}}\alpha (t_{0})z_{s}^{2}\right\}$$

(3) $$=\cos\left\{2n\left[z_{s}+\frac{p\omega_{m}c}{2n\alpha (t_{0})}\right]k+p\omega_{m}\left[t_{0}-\frac{\omega (t_{0})}{\alpha (t_{0})}\right]-2\frac{n^{2}}{c^{2}}\alpha (t_{0})z_{s}^{2}\right\}.$$

The first term is proportional to the wavenumber, *k*. It can thus be expected that the $p^{\text{th}}$ harmonic will appear axially shifted by a distance $\Delta z_{m}=p\frac{\omega_{m}c}{2n\alpha (t_{0})}$ . The remaining terms can also contribute a phase that may have a modest, indirect, dependency on wavenumber. The angular acceleration, α(t) , is constant for an ideal linear swept source laser. In practice, for all harmonics but the fundamental image (p=0) , variability in the frequency sweep will result in an axial blur akin to dispersion.

By moving the direct image of the sample outwith the axial range, the phase modulation can be used to precisely position the non-conjugate and conjugate images to avoid any overlap. As can be seen in Fig. 1(c), the image of a curved object as the human retina will always overlap with its conjugate. However, electro-optical modulation is sufficiently fast to dynamically adjust the frequency shift, and thus depth, during the scan. This allows us to artificially flatten the 1^st^-harmonic image of the curved retina (Fig. 1(d)). It is then feasible to single out the non-conjugate first-harmonic image to digitally reconstruct a curved retina (Fig. 1(e)).

We need to overcome several challenges for a practical implementation. Firstly, the coherence length of the swept-source laser must extend beyond the first order to ensure interference contrast. Only recently this has become within reach with the advent of MEMS-VCSEL lasers. These combine a fast sweep rate with a coherence length that is measured in tens of meters. Secondly, the power in the first harmonic is approximately proportional to the signal-to-noise ratio. In the case of sine-wave phase modulation, $A_{m}\sin(\omega_{m}t)$ , the amplitude of the *p*^th^-harmonic is given by the Jacobi-Anger expansion as $J_{p}(A_{m})$ . It can be numerically verified that for $A_{m}\approx 1.841\;\text{rad}$ this reaches a maximum of 0.582 (−4.7 dB in intensity) for the first harmonic. Finally, since the frequency sweep of such sources is not perfectly linear, the interference should not be sampled uniformly in time but rather synchronous with the laser frequency, k(t)=ω(t)/c . This well-known problem is generally addressed by triggering the acquisition of each sample based on the signal of an auxiliary Mach-Zehnder interferometer. However, we found that this is insufficient to adequately correct the image of harmonics. Variability in the sweep-rate, $\alpha (t)=\alpha_{0}+\Delta \alpha (t)$ , translates into a virtual delay of the reference beam by $p\frac{\omega_{m}c}{2n\alpha_{0}}\left[\frac{1}{1+\Delta \alpha (t)/\alpha_{0}}-1\right]\approx -p\frac{\omega_{m}c}{2n\alpha_{0}^{2}}\Delta \alpha (t)$ . In the next section, we will show how the resultant image blur can be avoided by digital correction of the measured spectral components.

## 3. Methods

### 3.1. Working principle

The opto-electronic reference arm plays the role of the mechanical delay stage. By leveraging the high temporal coherence of MEMS-VCSEL swept sources, we operate within a regime where the optical signal bandwidth surpasses acquisition electronics. To selectively place the sample within the electronic bandwidth, we employ a frequency shifting scheme using a fiber-coupled opto-electronic phase modulator driven by high-frequency voltage. The rapid phase modulation leads to the generation of harmonics separated by integer multiples of the modulation frequency.

Assuming a linear sweep, we can see that the shift coefficient, which can be defined as α=BR/D where *B* is the sampled frequency bandwidth of the source, *R* is the sweep rate, and *D* is the duty cycle of the sweep. The nominal values of our swept-source are B=160THz corresponding to a wavelength range of 95nm , at a central wavelength of 1060nm , duty cycle of 50 %, and a sweep rate of 400kHz . This gives us a shift coefficient of 7.4 mm/GHz. The phase modulator can support modulation of up to 10 GHz, providing a theoretical axial tuning range of 74mm . In practice, the axial range is limited by the dispersion caused by the non-linearity of the sweep. As discussed in section 4.2, this highlights the growing need for linear-in-k swept sources.

### 3.2. System design

To demonstrate its capabilities, We adapt two commercial OCT systems. A conventional lens-based system (Optos, Optos OCT SLO), capable of imaging with a 20μm resolution across a widefield 12mm lateral field-of-view; and a mirror-based system capable of ultra-widefield imaging, which we have reduced to a widefield 12.5mm lateral field-of-view (Optos, Silverstone). We use the OCT SLO system to image the convex surface of a biological sample (grape), and the Silverstone to measure a model eye. All images throughout the paper are an incoherent average of ten.

**Fig. 2.**
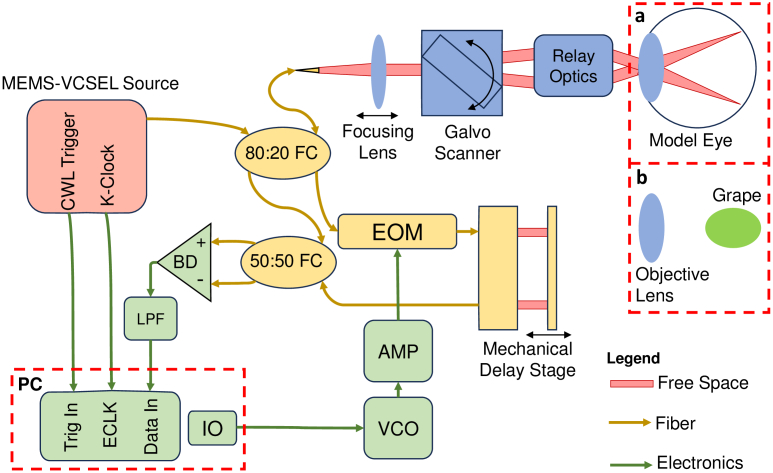
Detailed system diagram for harmonic OCT using electro-optical modulation. The reference arm contains the electro-optical modulator (EOM) for fast depth adjustments, as well as a slow mechanical delay stage for coarse adjustments of the optical path difference prior to the experiment. **a.** Sample arm configuration for imaging of the model eye. **b.** Sample arm configuration for imaging of the grape. An objective lens substitutes the ocular lens. LPF: low-pass filter, BD: balanced detector, FC: fiber coupler, VCO: voltage-controlled oscillator, AMP: amplifier, IO: input/output.

The full system diagram for both experiments is depicted in Figure 2. The systems have been retrofitted with a MEMS-VCSEL swept source (Thorlabs, SL104071) operating at a central wavelength of 1060nm , with an effective bandwidth of 95nm , 400kHz sweep rate, and 50% duty cycle. During the 1.25μs A-scan, we acquire 640 samples at a peak k-clock sample rate of 550MHz . This corresponds to an axial imaging depth of 2mm at a 400kHz A-scan rate. The source beam was split into a sample and reference path using a custom 80:20 fiber-coupler (OZ Optics, A10765004). The sample beam is collimated using a lens fixed on an axial translation stage, then scanned across the lateral field-of-view using a pair of galvanometers (Cambridge Technology, Model 6210H) before being relayed onto the cornea and focused onto the retina. The reference arm comprises of an electro-optical modulator (Jenoptik, PM1064) exhibiting a half-wave voltage of 6V , as well as a custom adjustable delay stage. The electro-optic phase modulator is driven with a voltage-controlled oscillator (Crystek, CRBV55BE-1000-2000) capable of generating a voltage in the 1-2 GHz radio-frequency range, at a nominal power of 7.9mW , the oscillator frequency is controlled with an analogue voltage produced with a multi-function IO board (National Instruments, PCIe-6374), as such it can be adjusted continuously during imaging. When testing the long range capability of the system, we instead used a wideband frequency synthesizer evaluation board (LMX2582EVM) with a frequency range of 5.5 GHz, albeit without continuous control. The radio-frequency signal is amplified using a radio-frequency gain block (Analog Devices, ADL5611) providing a fixed gain of 22.2 dB. The reference and sample arms interfere in a fused, single-mode fiber coupler (Thorlabs, TW1064R5A2A), the optical signal is then recorded using a high-frequency balanced photodetector (Thorlabs, PDB481C-AC) with a bandwidth of 1 GHz, the analogue signal is then filtered by an anti-aliasing filter with a cut-off frequency of 200MHz . The resultant signal is digitised using a digital data acquisition card (Alazartech, ATS9373), that can sample at an irregular external clock signal. By using the integrated Mach-Zehnder interferometer, known as the *k-clock*, we are able to sample at regular wavenumber intervals.

### 3.3. Numerical dispersion compensation

The detected spatial frequency is inversely proportional to the angular acceleration, $\alpha (t)=\alpha_{0}+\Delta \alpha (t)$ . Any deviation from the nominal, $\alpha_{0}$ , will thus result in a blurred image similar to dispersion.

Regardless of the source of the dispersion, it can be computationally compensated for as long as we can determine the phase error ϕ(k) . The iterative optimization approach for determining the phase error works well for material dispersion because it can be consistently described by a low-order polynomial. However, we found that when it is determined by the sweep non-linearity function, it may take on a much more complex shape. We therefore resort to a non-parametric method for estimating the phase error.

#### 3.3.1. Wigner-Ville distribution

A signal processing approach for numerical dispersion compensation via the short-time Fourier transform has been used to compensate for motion-induced dispersion in full-field OCT [33]. By investigating the time-frequency behaviour of the interferogram using this technique, it is possible to determine the phase-error directly from image data.

The Wigner-Ville distribution (WVD), W , provides much finer resolution in both time and frequency than the short-time Fourier transform [34]. The WVD of a mean-zero signal, sampled in wavenumber is

(4) $$S_{W}(k,z)=W[f(k)]=\int_{-\infty }^{\infty }R_{ff}(k,\kappa )e^{-iz\kappa }d\kappa =\int_{-\infty }^{\infty }f\left(k+\frac{\kappa }{2}\right)\bar{f\left(k-\frac{\kappa }{2}\right)}e^{-iz\kappa }d\kappa ,$$

where $R_{ff}(t,\kappa )$ is the instantaneous autocorrelation function.

The downside of this distribution is the generation of so-called *cross terms*, which arise in cases of signals which contain multiple frequency components. These cross terms can make interpreting the distribution difficult, for this reason the Pseudo Wigner-Ville distribution (PWVD) is often used in practice

(5) $$S_{W_{P}}(k,z)=W_{P}[f(k)]=\iint_{-\infty }^{\infty }h(k-k^{\prime })H(z-z^{\prime })S_{W}(k^{\prime },z^{\prime })dk^{\prime }dz^{\prime }.$$

The cross terms can be suppressed by low-pass filtering the Wigner-Ville distribution in frequency and time using separate filter kernels h(k) and H(z) which we have chosen to be Gaussian. The pseudo Wigner-Ville distribution generally exhibits better time-frequency resolution than the short-time Fourier transform, even when processing complex multi-component signals [35].

The PWVD of an OCT interferogram at a given wavenumber can be interpreted as an A-scan containing only a subset of spectral information around that wavenumber. In the absence of any dispersion, we would expect the distribution to be invariant with wavenumber. In order to estimate the phase error, we track the translation of the A-scan along *z* as a function of wavenumber. The phase error can be estimated up to a constant term, *C*, by integrating the displacement with respect to wavenumber [33], i.e.,

(6) ϕ(k)≈∫Δz(k)dk+C.

## 4. Results

### 4.1. Numerical dispersion compensation of harmonic images

The non-linear frequency sweep causes spectral broadening of the frequency-shifted images even when linear-in-k sampling using a hardware k-clock is implemented. We use a time-frequency analysis technique to estimate the phase error directly from the image and use it to perform numerical dispersion compensation. This technique is agnostic towards the source of the phase error so can also be used to correct for material dispersion and motion.

The effectiveness of the proposed numerical dispersion approach is investigated by simulating the signal formation process of an OCT system with a non-linear frequency sweep. The shape of the sweep was measured by recording the k-clock of a bench-top swept-source using a fast digitizer, calculating the spectrogram of the recorded signal, then fitting a fifth-order polynomial to the data.

The non-linear sweep vectors corresponding to the phase of sample and reference light are generated using the recorded polynomial. We simulate the frequency shift by simply adding a linear term to the reference arm phase. This approach assumes that there is no group velocity or higher order dispersion, as such, any spectral broadening can be attributed to the frequency shift.

**Fig. 3.**
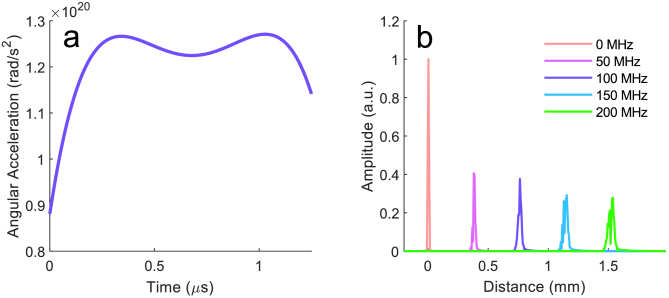
Variability of the sweep-rate. **a.** The angular acceleration, i.e., the rate of change of angular frequency with time, used for simulations is on the order of $1.2\times 10^{20}\;{\text{rad/s}}^{2}\approx 20\;\text{EHz/s}$ . This was estimated by recording the k-clock signal from the swept source. **b.** Simulated A-scans showing a single reflector. The broadening of the A-line is a result of the non-linear frequency sweep, despite linear-in-k sampling.

The interferogram is then generated by taking the cosine of the difference between the sample and reference phase vectors. The k-clock operation is modeled as a simple linear interpolation of the acquired interferogram, such that the data is expressed linearly with wavenumber. Following this, we process the interferogram in the standard way to obtain the simulated A-scans. The derivative of the simulated frequency sweep is shown in Fig. 3(a). The resultant A-scans (Fig. 3(b)) exhibit axial blurring with increasing frequency offset due to sweep dispersion.

**Fig. 4.**
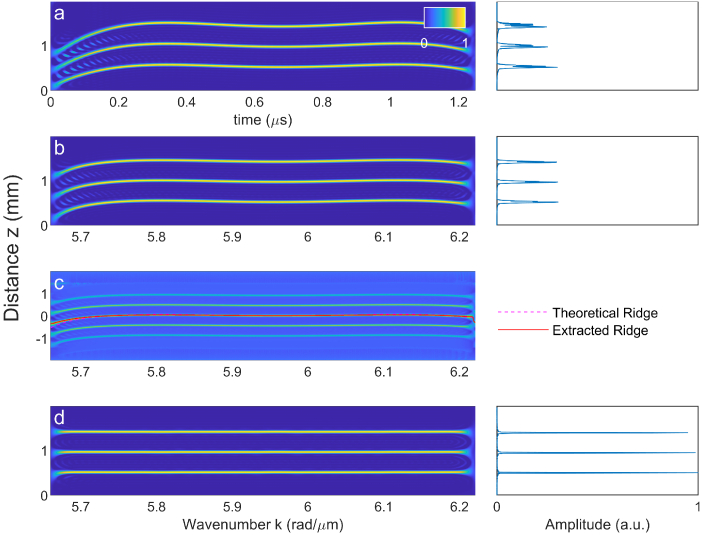
Numerical dispersion compensation by Pseudo Wigner-Ville Distribution (PWVD) analysis. **a.** PWVD of simulated interferogram sampled in time, and corresponding A-scan. Note the phase error is depth-dependent. **b.** PWVD of simulated interferogram sampled in wavenumber and corresponding A-scan. With the correct sampling, the phase error is no longer depth-dependent. **c.** Cross-correlation of PWVD, showing translation of the A-scan along depth. Both the theoretical and numerically-extracted ridge are shown. **d.** PWVD of interferogram after numerical compensation using extracted ridge, and corresponding A-scan. Note the apparent sample distance is no longer dependent on wavenumber.

We can use the Pseudo-Wigner-Ville Distribution (PWVD) to quantify and correct the phase error. Figures 4(a) and 4(b) show the PWVD of harmonic OCT both using linear-in-t and linear-in-k sampling. The sample consists of three discrete reflectors, allowing us to see how the phase error changes with depth. As expected, when sampling in t, there is a depth-dependent phase error. Resampling in k removes the depth dependence, however a residual error is still present in the interferogram.

We can estimate this phase error directly from the PWVD data, we do this by cross correlating one column of the PWVD with the rest of the distribution, allowing us to estimate the k-dependent translation of the image during the sweep. We can then obtain the corrective phase according to Eq. (6).

The cross-correlation of the PWVD, along with the extracted ridge is shown in Fig. 4(c). The theoretical ridge based on the sweep non-linearity Δα(t) is also shown. The two ridges are in strong agreement across most of the spectrogram, only at the edges the lines are not as well-resolved. This is not expected to have an substantial impact on the A-scan, as spectral information near the edges of the interferogram is generally windowed out prior to taking the Fourier transform. As a result, the A-scan is reconstructed at the theoretical resolution limit, using only image data, without requiring any prior calibration measurements.

**Fig. 5.**
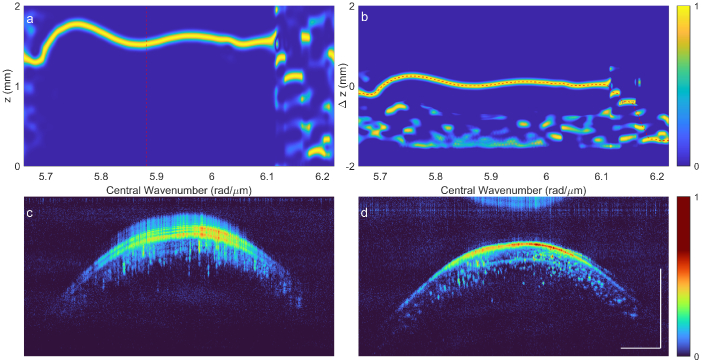
Experimental demonstration of effectiveness of numerical dispersion compensation for Harmonic OCT. **a.** Pseudo Wigner-Ville distribution of an A-line, we can see the position of the reflection move with the sweep, indicating a phase error in the interferogram. The red dashed line denotes the band-pass filtered A-scan used for cross-correlation. Each column was normalised individually for visual clarity. **b.** Cross-correlation of selected band-pass filtered A-scan with the full Wigner-Ville spectrum, the apparent displacement Δz of the sample is shown with a red dashed-line. **c.** B-scan of harmonic image prior to dispersion compensation, the blurring is primarily due to the non-linear laser sweep. **d.** B-scan after dispersion compensation has been applied. Scale bars are 1mm .

Figure 5 shows the process of dispersion correction using the cross-correlation of the pseudo Wigner-Ville distribution using real-world data. The ridge in Fig. 5(b) is extracted using a penalized backward-forward greedy algorithm (*tfridge* in Matlab). The resultant ridge represents the axial position fluctuations during the sweep. The integral of this ridge therefore represents the phase error. We can see that the ridge is not visible across the whole sweep. This is likely due to the shape of the laser spectrum which drops off at the start and end of the frequency sweep.

Our basic Matlab implementation (see Code availability) allowed us to compute the phase error in under 10 seconds on a desktop CPU (Intel i7-10850H at 2.7 GHz). We reuse the phase from an initial B-scan for coarse-grained correction during live imaging. The PWVD algorithm is used post-acquisition to perform fine correction and recover optimal resolution. While a basic implementation was deemed sufficient for demonstration purposes, we believe that real-time feedback is well within reach of optimized low-level codes.

### 4.2. Extended range imaging

The maximum shift distance is much greater than the axial imaging range of the system. As such, the opto-electronic reference arm could completely replace the mechanical reference arm. We investigate the long-range performance of the frequency shifting scheme by detuning the optical path difference using a mechanical translation stage, then compensating with the phase modulator to keep the harmonic image in the center of the image region.

**Fig. 6.**
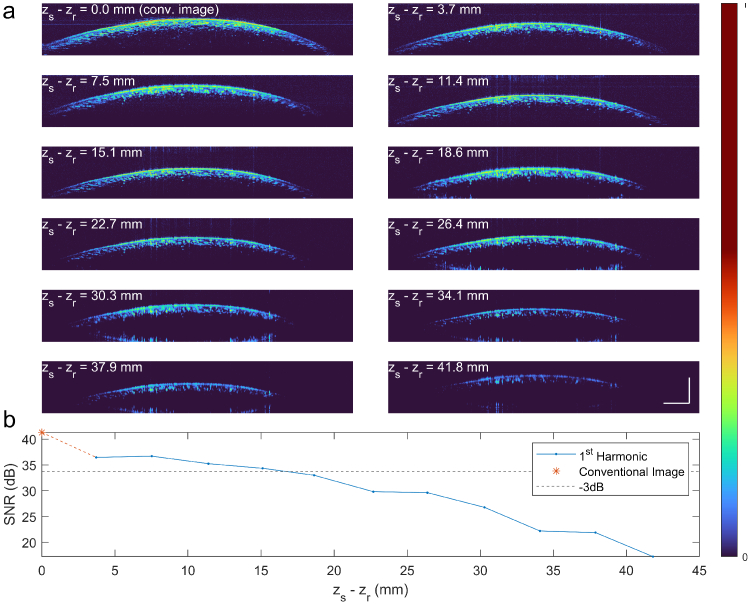
Assessment of long axial range shifting capability of the opto-electronic reference arm. **a.** B-scans taken at increasing values of $z_{s}-z_{r}$ show how high image contrast is maintained beyond the centimeter range. It decreases gradually until it is lost at around 40mm . **b.** The SNR as a function of the path difference $z_{s}-z_{r}$ . The first point corresponds to the conventional OCT image. Up to 15mm , the signal-to-noise relative to the conventional image remains above the -3 dB threshold. Scale bars are 1mm .

Figure 6 shows images at distances from 0 to 41.8mm . A gradual SNR roll-off as a function of Δz can be noted. Considering a -3 dB reduction as the practical threshold, the effective depth range is 17mm , a factor of 8 beyond the original axial imaging range of the source. The additional SNR losses can be explained by the imperfect phase error reconstruction. The SNR difference between the real image and a low Δz harmonic image is found to be -4.9 dB, which is in reasonable agreement with the theoretical value of -4.7 dB.

### 4.3. Sample flattening

Widefield OCT demands rapid depth adjustments during a B-scan in clinical practice. Typical samples curve quickly outwith the axial range of conventional OCT systems. Fast electro-optical adjustment of the axial range allowed us to track highly curved surfaces without sacrificing scan speed and with minimal loss in signal.

**Fig. 7.**
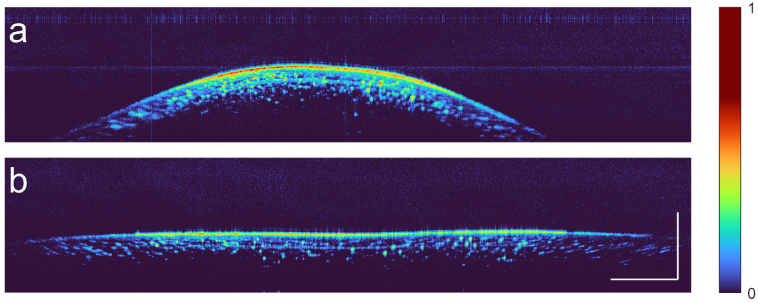
Experimental demonstration of the flattening of a convex surface (a grape). **a.** Conventional OCT image. **b.** High-contrast, flattened, harmonic image, acquired by electro-optically tracking the surface and compensating numerically for phase errors. Scale bars are 1mm .

**Fig. 8.**
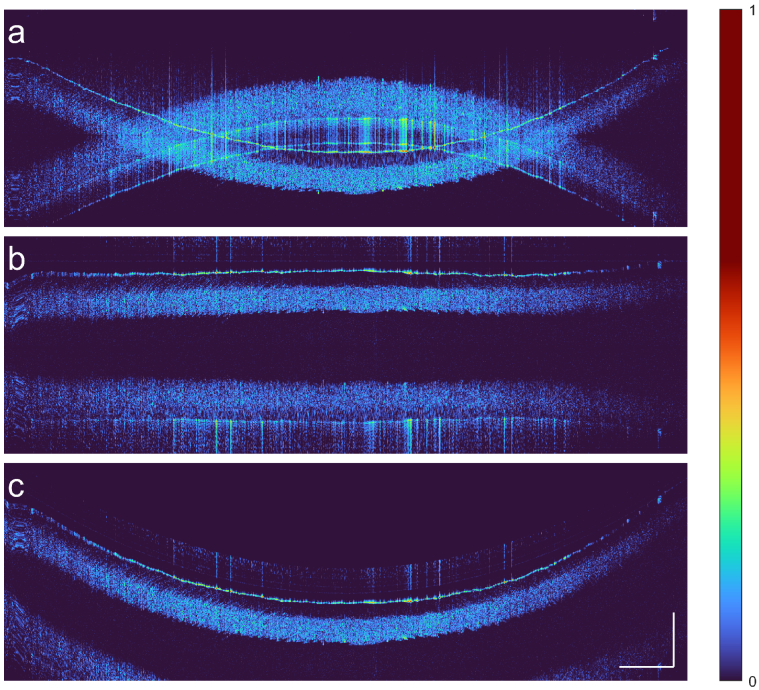
Experimental demonstration of electro-optical-digital flattening of the concave retina. **a.** The Harmonic image without fast depth-range extension. The field-of-view width without overlap is restricted to about 3mm near its center. Overlap with the complex conjugate image prevents imaging beyond a depth of approximately 1mm , yet the curved retina spans about 12.5mm×4mm . **b.** By tuning the frequency offset, the A-scan position is adjusted to cancel out the curvature of the sample and disentangle the desired image from the conjugate artifact. **c.** Harmonic image following numerical curvature reconstruction. Although the complex conjugate image can be computationally removed prior to reconstruction, it is purposely left in to show that it does not overlap with the true image. The conjugate ambiguity has been resolved without resorting to full-range techniques.

As a first demonstration, we imaged the surface of a small, highly-curved, grape. Figure 7(a) shows the conventional OCT image. Electro-optical modulation creates a harmonic image at a depth that can be adapted to the surface at the microsecond scale. This comfortably enabled a B-scan rate of 100Hz , each with 2,000 A-scans taken at 400kHz . As can be seen from Fig. 7(b), this not only flattens the image, it also improves the contrast in the periphery. Numerical dispersion compensation is used to cancel the phase errors induced by irregularities in the laser sweep rate. In tandem, Hybrid OCT extended the field-of-view by approximately 1mm on both sides. Figure 8 shows B-scans of an $81^{\circ }$ , measured as the input angle of the lens, section of a realistic model eye. As can be seen in Fig. 8(a), the 12.5mm×4mm scan area of the laser is too large to avoid overlap with the conjugate image. In this case, the field-of-view would have to be restricted to the central 3mm . Rather than resolving the complex conjugate using computational methods such as dispersion-encoded full-range [36,37], we simply disentangle the two images during acquisition by flattening the retina (Fig. 8(b)). We implemented parabolic flattening as a demonstration. In general one could adaptively locate the surface based on a prior scan [25,26,31,32]. The true image is visible in the top-half of the depth range, while the conjugate copy can be seen in the bottom range. The latter exhibits a notable dispersion blur due to the inverse effect of numerical dispersion compensation on the conjugate image. If desired, the physical shape can be digitally reconstructed over the entire field-of-view as depicted in Fig. 8(c). Harmonic OCT effectively extends the field-of-view to 12.5mm , without compromising on the fast 400kHz A-scan rate.

Hybrid OCT combines the speed and contrast of swept-source OCT with the clinical advantages of widefield images. The former are essential for *in vivo* applications such as retinal imaging, while the latter is key for early diagnosis and intervention.

## 5. Discussion and conclusion

Hybrid Optical Coherence Tomography enables widefield imaging with the speed and contrast demanded of clinical applications. To the best of our knowledge, Hybrid OCT is the first use of electro-optical modulation for microsecond-scale depth selection. It accomplishes high-contrast imaging through the confluence of three key ideas. Firstly, the electro-optical modulation of the reference arm virtually delays each harmonic by a controllable time, corresponding to a distance in the sample. Secondly, the advent of long coherence-length MEMS-VCSEL swept-source lasers enables high-contrast self-interference well beyond the depth of any clinical sample. Finally, numerical dispersion compensation must be used to eliminate the inevitable phase errors in the harmonic components due to imperfections in the laser sweep. We demonstrated how this can effectively extend the imaging range by a factor of 8.

Microsecond-scale electro-optical depth adjustments for individual A-scans are possible without slowing down the imaging process. We showed how this enables fast tracking of highly-curved surfaces such as the retina. We demonstrated intra-B-scan depth adjustment within the 2mm axial imaging range of the system, at the high A-scan rate of 400kHz . This allowed us to decouple the lateral and axial range in widefield retinal OCT. We showed that a wide field-of-view of $81^{\circ }$ input angle at the lens can be achieved with a fast swept source. The axial range requirement is only defined by the thickness of the retina, not by the overall size of the eye in the image. This allows us to take more data at large fields-of-view without increasing the imaging time, nor imposing additional requirements on the data acquisition chain.

The axial imaging range of Harmonic OCT is effectively determined by the number of k-clock samples per sweep or A-scan, and not at all by the much longer coherence time of the VCSEL source. The current 2mm axial imaging range could be increased proportional to the sample rate provided that the data-acquisition card can handle the faster clock signal. As swept-sources sweep rates are on track to surpass 1MHz in the near future, the bandwidth of the data acquisition electronics is bound to become an important design constraint. While electro-optical modulation increases the overall complexity of the SS-OCT somewhat, it facilitates efficient use of the limited data acquisition bandwidth.

We showed how the unavoidable variability in the laser sweep rate induces phase errors in the harmonic components. Although we demonstrated how this can be compensated for using numerical dispersion correction, excessive optical path differences would be filtered out to avoid aliasing. This highlights the need for linear-in-k sources for long-range harmonic imaging.

Harmonic OCT is compatible with existing dispersion-encoded full-range approaches for removing the conjugate image artefact [36,37]. Intriguingly, the sweep-induced dispersion could aid the dispersion-encoded full-range reconstruction process. The effect could be enhanced by tailoring the laser-sweep rate to cause the desired synthetic dispersion. This would eliminate the need for bulky and expensive dispersive glass to enable dispersion-encoded full-range.

Additionally, this scheme could be enhanced with more advanced surface tracking [26], potentially allowing for automatic flattening of complex surfaces. Harmonic OCT could speed up the contour-mapping as well as the imaging process, leading to decreased imaging time and improved image-quality. Similarly, adaptive axial motion tracking techniques could be integrated with the electro-optical modulation for real-time adaptive position and motion tracking [25,31,32].

In our current implementation, the phase modulation comes at the cost of a modest reduction in signal to noise ratio. We showed how for sine-wave modulation the signal in the harmonic is at best 4.7 dB (37%) lower than that of conventional swept-source OCT. To put this in context, time-domain OCT typically has a 30 dB lower signal-to-noise ratio [14]. Even so, if need be, the efficiency can be improved beyond that of sine-wave modulation. The ideal saw-tooth modulation could be closer approximated using high-frequency electro-optical modulation, or through a pair of acousto-optic modulators. Do note that the bandwidth of the current generation of acousto-optic modulators would severely restrict the shifting range.

In its present form, Harmonic OCT is specific to Swept-Source OCT. This modality offers excellent signal-to-noise contrast. Although the typical SNR roll-off performance of Spectral-Domain OCT cannot match that of Swept-Source OCT, it may achieve ultrahigh axial resolution. It is not immediately obvious how Harmonic OCT can be practically extended to other modalities.

In conclusion, Harmonic Optical Coherence Tomography brings widefield imaging within reach of clinical OCT. Imaging in patients demands the high speed and contrast of swept-source OCT systems, while widefield imaging raises the potential of early detection of disease. We showed how Harmonic OCT extends the field-of-view with a modest impact on signal strength and without reducing the scan speed, even for a novel 400kHz swept source. Electro-optical modulation will become ever more important as commercial systems move towards megahertz scan rates. Interestingly, we found that irregularities in the laser sweep rate induce a novel type of dispersion in the harmonic image. We showed that these phase errors can be readily compensated for by numeric means. Using Harmonic OCT, we demonstrated an effective 4-fold extension of the field-of-view with an 8-fold extension of the axial range. Any OCT system with a temporally coherent swept-source can be readily transformed into a Harmonic OCT system. It is straightforward to integrate an off-the-shelf electro-optical modulator into the reference arm alongside the corresponding software. We anticipate that its application in the clinic will aid the early detection of ocular disease and improve patient outcomes.

## Data Availability

The algorithm, as well as the data visualization, is implemented in Matlab. The complete source code with examples is openly available as a Git repository under the MIT License [38].
